# Biological sex influences psychological aspects of the biopsychosocial model related to chronic pain intensity and interference among South Korean patients with chronic secondary musculoskeletal pain in rheumatic diseases

**DOI:** 10.3389/fpsyg.2023.1063164

**Published:** 2023-04-17

**Authors:** Hee Jun Kim, Timothy J. Meeker, Ju-Yang Jung, Ji-Won Kim, Hyoun-Ah Kim

**Affiliations:** ^1^School of Nursing, The George Washington University, Washington, DC, United States; ^2^Department of Biology, Morgan State University, Baltimore, MD, United States; ^3^Department of Rheumatology, Ajou University School of Medicine, Suwon-si, Republic of Korea

**Keywords:** chronic pain, pain catastrophizing, depression, rheumatic disease, biopsychosocial, musculoskeletal, sex differences

## Abstract

**Introduction:**

Pain is a prominent contributor to negative personal and social outcomes, including increased disability and mortality, in many rheumatic diseases. In the Biopsychosocial model of chronic pain, psychological and social factors share roles with the biology of the injury in determining each patient’s pain and suffering. The current study explored factors associated with clinical pain intensity and interference among patients with chronic secondary musculoskeletal pain in rheumatic diseases.

**Methods:**

In total, 220 patients experiencing chronic secondary musculoskeletal pain participated. Biological factors (age, biological sex, pain condition, pain duration, pain sensitivity, and comorbidity), socio-economic factors, psychological factors (pain catastrophizing and depressive symptoms), and pain intensity and interference were measured. Descriptive, multivariable linear regression and partial correlation analyses were conducted. Subgroup analysis by sex was conducted to examine differences in how different factors affect the pain experience.

**Results:**

The mean age of the participants was 52.3 years (*SD* = 12.07) and ranged from 22 to 78. Average pain intensity was 3.01 (0–10 scale) and average total pain interference score was 21.07 (0–70 scale). Partial correlation found positive correlations between pain intensity and interference with depression (intensity: *R* = 0.224; *p* = 0.0011; interference: *R* = 0.351; *p* < 0.001) and pain catastrophizing (intensity: *R* = 0.520; *p* < 0.001; interference: *R* = 0.464; *p* < 0.001). In males, pain condition (*β* = −0.249, *p* = 0.032) and pain catastrophizing (*R* = 0.480, *p* < 0.001) were associated with pain intensity. In males, the simple correlation between pain intensity and depression (*R* = 0.519; *p* < 0.001) was driven by pain catastrophizing. In females, pain catastrophizing (*R* = 0.536, *p* < 0.001) and depressive symptoms (*R* = 0.228, *p* = 0.0077) were independently associated with pain intensity. Age (*β* = −0.251, *p* = 0.042) and pain catastrophizing (*R* = 0.609, *p* < 0.001) were associated with pain interference in males, while depressive symptoms (*R* = 0.439, *p* < 0.001) and pain catastrophizing (*R* = 0.403, *p* < 0.001) were associated with pain interference in females. Again, in males, the simple correlation between pain interference and depression (*R* = 0.455; *p* < 0.001) was driven by pain catastrophizing.

**Discussion:**

In this study, females were more directly affected by depressive symptoms than males, regarding pain intensity and interference. Pain catastrophizing was a significant factor influencing chronic pain for both males and females. Based on these findings, a sex-specific approach to the Biopsychosocial model should be considered in understanding and managing pain among Asians with chronic secondary musculoskeletal pain.

## 1. Introduction

Chronic pain affects a significant number of patients worldwide. Estimated prevalence of chronic pain ranged from 35.0 to 51.3% in the United Kingdom (Fayaz et al., 2016), and 11 to 40% in the US (Dahlhamer et al., 2018). In South Korea, nationwide prevalence of chronic pain in adults has been rarely reported. However, one study reported prevalence in older adults of 87.8% for females and 63.8% for males (Jung-Choi et al., 2014). Chronic pain, defined as pain lasting more than 3 months, is frequently refractory to treatment (Jensen, 2016); meta-analyses indicate that only about half of patients experience clinically meaningful pain relief from pharmacological therapies (Bjordal et al., 2007; Machado et al., 2015). The new *International Classification of Diseases (ICD-11)* introduced the concept of chronic primary and secondary pain, stating that chronic pain should be regarded as a condition in its own right, rather than being assigned by the underlying diagnosis (Perrot et al., 2019). This new definition integrates the biomedical, psychological, and social axes that comprise the complex experience of chronic pain, recognizing its independent impact on functioning. Treatments are frequently undermined by various psychological and social factors which influence the pain experience. Comprehensive and accurate assessment of chronic pain and long-term evidence-based treatment plans aimed at factors influencing pain outcomes are necessary to better care for patients with chronic pain.

Chronic pain is a complex, subjective experience influenced by multiple factors. The Biopsychosocial model of pain experience is the dominant framework for understanding the complexity of chronic pain (Fifield et al., 1991; Fordyce, 1994; Sullivan et al., 2022). The Biopsychosocial model seeks to acknowledge and measure the combined influence of sociological, psychological, and biological factors influencing the pain experience. Previous studies on the role of age and biological sex have shown that older patients and women experience chronic pain with greater intensity (Mills et al., 2019). An individual’s degree of sensitivity to painful stimuli has also been associated with chronic pain. Differences in experimental pain sensitivity were reported in patients with chronic pain (Cardoso et al., 2016), and higher experimental pain sensitivity was associated with greater clinical pain intensity among elderly patients with knee osteoarthritis (Cruz-Almeida et al., 2014). Furthermore, greater mechanical cutaneous pain sensitivity was associated with clinical pain intensity in patients with chronic knee osteoarthritis (Alabas et al., 2013). Including quantitative sensory testing (QST) assessment in chronic musculoskeletal pain may allow the patient to be categorized into phenotypic subgroups (Uddin and MacDermid, 2016).

In terms of psychosocial factors, depression, anxiety, sleep quality, quality of life, and pain catastrophizing are all reported to be associated with the chronic pain experience, impacting on both pain intensity and pain interference (Kim et al., 2020). In a racial/ethnic comparison study, depression and pain catastrophizing were significant factors explaining higher pain intensity among Asians compared to non-Hispanic Whites (Kim et al., 2019). Pain catastrophizing was also found to have a significant mediating effect on the relationship between depression and pain intensity in Asians with chronic pain (Kim et al., 2021). Socio-economic background was reported to be inversely related to chronic pain; severe pain intensity and greater level of pain-related disability were associated with socio-economic deprivation; those who have lower levels of education and perceived economic status were more likely to experience chronic pain than those with higher levels of education and economic status (Mills et al., 2019).

Asians have been under-represented in pain research in the US; evidence is lacking on chronic pain experiences of Asians due to their minimal sampling in chronic pain studies (Kawi et al., 2019). Without considering social and cultural aspects, chronic pain cannot be understood nor effectively managed (Bostick et al., 2021). Racial/ethnic and cultural impact on biopsychosocial aspects of pain experience in patients with chronic secondary musculoskeletal pain has been reported in general populations (Orhan et al., 2018), however, evidence on Asians on this matter is still limited. The purpose of this study was to examine concurrent influences of multidimensional correlates of chronic secondary musculoskeletal pain among Korean patients with various rheumatic disease, as an example of an Asian subpopulation. We also aimed to evaluate biological sex differences in how biopsychosocial factors affect chronic secondary musculoskeletal pain in Korean culture. We hypothesized that psychological factors will be the most significant factors influencing pain experiences (pain intensity/interference) compared to other social/biological factors, and there will be significant differences in those associations between pain experiences and biopsychosocial factors by biological sex.

## 2. Materials and methods

### 2.1. Design

An exploratory, cross-sectional design was used for this study.

### 2.2. Participants

The participants recruited for this study were patients with chronic musculoskeletal pain. The inclusion criteria were as follows: (1) age > 18 years, (2) able to communicate verbally and in written form and to provide written informed consent, and 3) chronic musculoskeletal pain (≥3 months). Exclusion criteria were: (1) serious medical conditions (e.g., uncontrolled hypertension, cardiac diseases), (2) peripheral neuropathy, (3) diagnosed with fibromyalgia or Systemic Lupus Erythematosus, (4) cognitive impairment, (5) daily use of opioids, or (6) hospitalization within the preceding year for psychiatric illness.

### 2.3. Procedure/ethical consideration

Participant recruitment was conducted in a rheumatic outpatient clinic at a hospital in a province in South Korea. Patients who met the inclusion/exclusion criteria were informed about the study by clinicians. If patients were interested in participating in the study, a research assistant approached the potential participants and explained the study process and purposes. Once they agreed to participate, written informed consent was obtained before data collection. Participants were assured that they could withdraw from the study if they wished at any time. The data collection procedure started with pain sensitivity measurement, conducted by a trained research assistant, and then participants completed self-reported questionnaires. To reduce missing data, the research assistant reviewed the written questionnaires after they were completed and answered any questions raised by the participants. Participants who completed the study were reimbursed with an $18 online gift-card. The study protocol and questionnaires were reviewed and approved by the Ajou University Hospital IRB (AJIRB-MED-SUR-21-319) prior to the data collection. Data was collected from September to October 2021.

### 2.4. Measures

We measured biological factors, including age, biological sex, diseases associated with pain, pain duration, comorbidity, and experimental pain sensitivity. Psychological factors include depressive symptoms and pain catastrophizing that was previously reported associated with chronic pain, especially among Asians (Kim et al., 2019, 2021). For social factors, we included level of education, perceived economic status, religion, type of residency, and marital status.

#### 2.4.1. Quantitative sensory testing

Experimental pain sensitivity was measured using a QST procedure (Rolke et al., 2006). Pressure pain threshold, tactile threshold, mechanical cutaneous pain threshold, and temporal summation of painful cutaneous mechanical stimuli were included. Pressure pain threshold was measured using a digital pressure algometer (FPX25, Wagner Instrument, Greenwich, CT, United States); patients were asked to say “now” when they felt “pain or discomfort” when the examiner applied a pressure with a 30 kPa/s speed (increments of 30–50 N/m^2^ per second). We tested the forearm area, avoiding any painful areas. In total, five trials were conducted using adjacent skin areas. First trial data were excluded in data analyses. Mechanical cutaneous pain sensitivity was measured using monofilaments (Von Frey Filament, North Coast Medical Inc. Touch-Test Sensory Evaluator, United States). Participants were asked to say “now” when they first felt touch (tactile threshold) change to “pain or discomfort” by increasing the force exerted by the monofilament (from 0.008 to 300 g) to assess mechanical cutaneous pain thresholds. Temporal summation of painful cutaneous mechanical stimuli was assessed using the 300 g filament. One stimulus was applied, and the patient was asked to rate the pain from 0 to 100 scale. Subsequently 10 stimuli, delivered every 2 s, were applied and patients were asked to rate their pain every trial. The difference between the first and the highest pain rating was calculated and taken as the summation rating (Greenspan et al., 2011; Goodin et al., 2014).

#### 2.4.2. Psychological factors

Pain catastrophizing was measured using the Pain Catastrophizing Scale (PCS) (Sullivan et al., 1995). The tool is a 13-item scale that assesses catastrophic thinking, including subscales of helplessness, rumination, and magnification, in response to pain. The PCS total score was calculated by summing the total item responses; higher scores on the PCS are indicative of greater pain-related catastrophizing. The Korean version of the PCS (K-PCS) used for this study has been previously validated in patients with chronic non-cancer pain (Cho et al., 2013). Cronbach’s alpha was 0.92 in this study.

The previously validated Patient Health Questionniaire-9 Korean version (PHQ-9 K) was used to measure depressive symptoms (Donnelly and Kim, 2008). The PHQ-9 is a self-administered questionnaire and includes nine items based on the nine diagnostic criteria for a major depressive episode in Diagnostic and Statistical Manual of Mental Disorders (DSM-5) (American Psychiatric Association, 2013). The tool has been validated in the Korean population previously (Shin et al., 2010), and the Cronbach’s alpha was 0.84 in this study.

#### 2.4.3. Pain intensity/interference

The Korean version of Brief Pain Inventory (BPI) was used to measure the level of chronic pain of the participants (Yun et al., 2004). This instrument examines both pain intensity and the level of pain interference due to chronic pain (Cleeland, 1991). The BPI uses an 11-point numeric rating scale (0 = “no pain” and 10 = “pain as bad as you can imagine”) to evaluate pain at its worst, least, average, and current severity over the past week. The BPI also evaluates the degree of interference with general activity, mood, walking, work, sleep, relations with others, and enjoyment of life due to pain, using a numeric rating scale (0 = “no interference” and 10 = “interferes completely”). Cronbach’s alpha for pain severity was 0.84, and pain interference was 0.93.

#### 2.4.4. Demographic and social factors

Age, biological sex, levels of education, perceived economic status, religion, marital status, and type of residency (e.g., living alone, living with children, etc.) were included. Chronic pain condition, duration, and comorbidities were also assessed.

### 2.5. Statistical analysis

Descriptive analyses were conducted to provide background information of the participants. Normality of the data was verified using histograms, normal probability plots, and skewness/kurtosis measures. Bivariate analyses were conducted to examine relationships between study variables (biological, social, and psychological factors) and the dependent variables (pain intensity and pain interference) using Pearson’s correlations. Subgroup analysis of multivariable linear regression was conducted to examine significant factors associated with pain intensity and pain interference by biological sex. Assumptions for regression analysis were checked and interdependence and linearity were evaluated through bivariate scatter plots. Scatter plots and the Levene’s test was used to determine homoscedasticity. The multivariable model included biological factors (age, sex, chronic pain condition, pain duration, comorbidity), social factors including education level, perceived economic status, religion, marital status, and type of residency, and psychological factors, including depressive symptoms and pain catastrophizing; these were entered into the regression models. All analyses were performed using Stata version 17.1.

For continuous and pseudo-continuous predictors without significant collinearity including age, depressive symptoms, pain catastrophizing, tactile threshold, mechanical pain threshold, area under the curve for temporal summation of mechanical pain, fold change in mechanical pain during temporal summation, pressure pain threshold at the forearm, and pain duration in a partial correlation model with pain interference, pain intensity or number of pain areas were used as the outcomes of interest. Partial correlation models were solved using the ppcor package in R 4.2.1 (Kim, 2015). This package solves the multivariable correlation problem by inverting the covariance matrix, solving all partial correlations simultaneously. This avoids the necessity of specifying a hierarchical model. For figures, we used the R package ggscatter to create bivariate scatterplots with corresponding 95% confidence curves for relationships of interest (Meeker et al., 2021). To control for testing multiple statistical hypotheses within each partial correlation model we used a Bonferroni correction for 45 possible tests (*p* < 0.0011).

## 3. Results

### 3.1. Participants characteristics

In total, 220 participants signed their informed consent and completed all study procedures. Descriptive statistics of the sample are presented in Table 1. The mean age of the participants was 52.3 years (*SD* = 12.07). The sample was 65.5% female and married (70%). A majority (82%) of the participants had at least a high school education. Sixty-six percent of participants indicated their perceived economic status as middle socioeconomic status (SES), while 25.6% indicated self-endorsed low SES. About half of the participants were living with children. In terms of chronic pain condition, 55.5% of the participants reported being diagnosed with rheumatoid arthritis, 23.6% with ankylosing spondylitis, and 14.1% with osteoarthritis. Thirty-seven percent of the participants reported having pain for more than 10 years, while 29.7% indicated that they had pain for 5–10 years. Twenty-eight percent of participants indicated they had at least one comorbid condition, whereas 60% of the participants reported that they did not have other diseases. The most common comorbid condition was hypertension (22.3%).

**Table 1:** Participants characteristics (*N* = 220).

Characteristics		
	N	%
Age (Mean/SD)	52.33	12.07
Biological sex		
Male	76	34.55
Female	144	65.45
Education		
≤ middle school graduate	41	18.72
≤ high school graduate	85	38.81
≥college graduate	93	42.47
Religion		
Yes	98	44.55
No	122	55.45
Marital status		
Married	154	70.00
Else (e.g., single, divorced, separation, bereavement)	66	30.00
Residency type		
Living alone	35	15.98
Living as a couple	52	23.74
Living with children	109	49.77
Else (e.g., living with parents, with friends)	23	10.50
Perceived economic status		
High	18	8.22
Middle	145	66.21
Low	56	25.57
Pain related disease		
Rheumatoid arthritis	122	55.45
Osteoarthritis	31	14.09
Ankylosing spondylitis	52	23.64
Else (e.g., osteoporosis, herniated disc)	15	6.82
Pain duration		
3 months to 1 years	20	9.13
1–5 years	52	23.74
5–10 years	65	29.68
10 years over	82	37.44
# of comorbidity		
None	132	60.00
1	62	28.18
2 or more	26	11.82

### 3.2. Depressive symptoms, pain catastrophizing, pain intensity, and pain interference

Table 2 presents the descriptive statistics of depressive symptoms, pain catastrophizing, pain intensity, and pain interference reported by the participants. The mean score of depressive symptoms was 5.62 (*SD* = 4.8). Fifty-one percent of participants reported no depression, while others were categorized as having mild (29.8%), moderate (11.0%), moderate–severe (6.4%), or severe depression (0.9%). The average pain catastrophizing score was 14.99 (*SD* = 11.05). Means of each subscale of the PCS were 7.2 (*SD* = 4.54) for rumination, 4.8 (*SD* = 5.07) for helplessness, and 3.0 (*SD* = 2.87) for magnification.

**Table 2:** Depressive symptoms, pain catastrophizing, and pain intensity/interference (*N* = 220).

Characteristics	Mean	*SD*
Depressive symptoms (PHQ total score)	5.62	4.82
Depression (categorical) (N/%)		
None	113	51.83
Mild	65	29.82
Moderate	24	11.01
Moderate–Severe	14	6.42
Severe	2	0.92
Pain catastrophizing		
Rumination	7.16	4.54
Helplessness	4.80	5.07
Magnification	3.00	2.87
PCS Total	14.99	11.05
Pain intensity		
Worst	4.96	2.69
Least	1.59	1.71
Average	3.24	1.85
Now	2.24	2.11
Average score (total)	3.01	1.74
Pain interference	21.07	16.75
Pain localization (N/%)		
Finger	161	73.2
Knee	128	58.1
Shoulder	106	49.1
Wrist	93	42.3
Ankle	73	33.2
Hand	67	30.5
waist	63	28.6
Toe	54	24.6
Elbow	53	24.1
Foot	48	21.9
Hipbone	44	20.0
Neck	36	16.4
Pelvis	30	13.7
Calf	23	10.5
Upper-arm	18	8.2
spine	18	8.2
Heel	17	7.7
Groin	14	6.4
Scapula	14	6.4
Back	13	5.9
Thigh	11	5.0
Forearm	10	4.6
Shin	9	4.1
Cheek	9	4.1
Else (e.g., Thorax, Head)	18	7.8

The average intensity of pain over the past week was 4.9 (*SD* = 2.69) for worst pain, 1.6 (*SD* = 1.71) for least pain, 3.2 (*SD* = 1.85) for average pain, and 2.2 (*SD* = 2.11) for current pain. The mean of the average scores of the four pain intensities was 3.00 (*SD* = 1.74). The average pain interference reported by the participants was 21.06 (*SD* = 16.75).

### 3.3. Pressure pain thresholds, mechanical cutaneous sensation thresholds, mechanical cutaneous pain thresholds, and temporal summation of mechanical cutaneous pain

The average of PPT was 8.12 Nm (*SD* = 2.11), 0.011 g (*SD* = 0.002) for mechanical cutaneous sensation threshold, and 0.021 g (*SD* = 0.002) for mechanical cutaneous pain threshold. The average of temporal summation of the mechanical cutaneous pain was 20.1 (*SD* = 14.5).

### 3.4. Depression, pain condition, and pain catastrophizing are associated with pain intensity and sex differences

We explored bivariate associations among the study variables; these results are presented in Supplementary Appendix 1. The first multivariable analysis included biological measures (age, pain-related disease type, pain duration, and comorbidity), socio-economic factors (level of education, perceived economic status, religion, marital status, and type of residency), and psychological factors, including depressive symptoms and pain catastrophizing, for both men and women (Table 3). The regression model for examining factors associated with pain intensity was significant, *n* = 216, *F*(21, 194) =8.79, *p* < 0.001, accounting for 43.2% of the variance in the pain intensity. The results indicated that pain-related disease type (*β* = −0.831, *p* = 0.030), pain catastrophizing (*β* = 0.071, *p* < 0.001), and depressive symptoms (*β* = 0.080, *p* < 0.001) were significantly associated with pain intensity.

**Table 3:** Multivariate analysis of biopsychosocial factors influencing pain intensity/interference (*N* = 220).

	Pain intensity	Pain interference
Biological sex (Reference: male)	0.184	2.879
Age (Reference: < 60 years)	−0.199	−2.449
Pain related disease (Reference: rheumatoid arthritis)		
Osteoarthritis	0.379	2.603
Ankylosing spondylitis	0.028	5.322*
Else (e.g., osteoporosis, herniated disc)	−0.831*	−5.784
Pain duration (Reference: < 1 year)		
1–5 years	0.172	1.972
5–10 years	−0.095	−4.600
10 years over	0.493	−0.989
# of comorbidity (Reference: 0)		
1	−0.012	−2.845
2 or more	−0.332	−4.661
Education (Reference: less than high school)		
≤ high school graduate	−0.167	−1.728
≥ college graduate	−0.254	−0.646
Perceived economic status (Reference: High)		
Middle	0.300	3.005
Low	0.600	6.925
Religion (Reference: no)	−0.117	0.610
Marital status (Reference: non-married)	0.052	0.125
Residency type (Reference: living alone)		
Living as a couple	0.265	8.016*
Living with children	0.612	4.975
Else (living with parents, with friends)	−0.110	−0.224
Pain catastrophizing	0.071***	0.564***
Depressive symptoms	0.081***	1.145***
Constant	0.781	−1.946
*R*^2^ (adjusted *R*^2^)	0.488 (0.432)	0.519 (0.466)
*F*	8.79***	9.95***

*p < 0.05, ***p < 0.001.

The multivariable subgroup regression models separated by biological sex were tested (Table 4). The regression model for examining factors associated with pain intensity for males was significant, *n* = 74, *F*(20, 53) =2.99, *p* = 0.0076, accounting for 35.3% of the variance in the pain intensity. In this model, being diagnosed with other rheumatic diseases (e.g., herniated disc) compared to rheumatoid arthritis (*β* = −2.374, *p* = 0.032) and higher pain catastrophizing (*β* = 0.053, *p* = 0.020) was significantly associated with higher pain intensity. For female participants, both psychological factors were significantly associated with pain intensity, indicating higher depressive symptoms (*β* = 0.073, *p* = 0.012), and pain catastrophizing (*β* = 0.077, *p* < 0.001) was associated with higher pain intensity. The model was significant, *n* = 142, *F*(20, 121) = 5.59, *p* < 0.001, accounting for 39.4% of the variance in the pain intensity.

**Table 4:** Subgroup analysis of biopsychosocial factors influencing pain intensity/interference by gender.

	Pain Intensity		Pain Interference	
	Males	Females	Males	Females
Age (Reference: < 60 years)	−0.226	−0.083	−9.627*	−0.563
Pain related disease (Reference: rheumatoid arthritis)				
Osteoarthritis	0.439	0.302	5.605	0.754
Ankylosing spondylitis	−0.032	0.067	4.968	3.591
Else (e.g., osteoporosis, herniated disc)	−2.374*	−0.542	−12.087	−5.083
Pain duration (Reference: < 1 year)				
1–5 years	−0.492	0.257	2.626	0.860
5–10 years	−1.086	0.045	−10.878	−3.099
10 years over	−0.175	0.592	−4.094	−1.324
# of comorbidity (Reference: 0)				
1	−0.324	0.025	0.477	−0.717
2 or more	−0.055	−0.572	1.025	−6.351
Education (Reference: less than high school)				
≤ high school graduate	−0.030	−0.012	1.538	−1.299
≥ college graduate	0.017	−0.225	4.110	−1.226
Perceived economic status (Reference: High)				
Middle	0.021	0.319	8.583	1.848
Low	0.627	0.636	15.645	6.095
Religion (Reference: no)	0.110	−0.172	4.006	−1.759
Marital status (Reference: non-married)	0.219	<−0.001	0.995	0.604
Residency type (Reference: living alone)				
Living as a couple	0.669	0.150	15.388	6.136
Living with children	1.016	0.477	7.875	4.442
Else (living with parents, with friends)	0.236	−0.243	−0.089	4.129
Pain catastrophizing	0.053*	0.077***	0.754**	0.483***
Depressive symptoms	0.107	0.072*	0.508	1.361***
Constant	1.139	0.833	−13.390	3.055
*R*^2^ (Adjusted *R*^2^)	0.530 (0.353)	0.480 (0.394)	0.623 (0.481)	0.501 (0.418)
*F*	2.99**	5.59***	4.39***	6.07***

*p < 0.05, **p < 0.01, ***p < 0.001.

**Figure 1:**
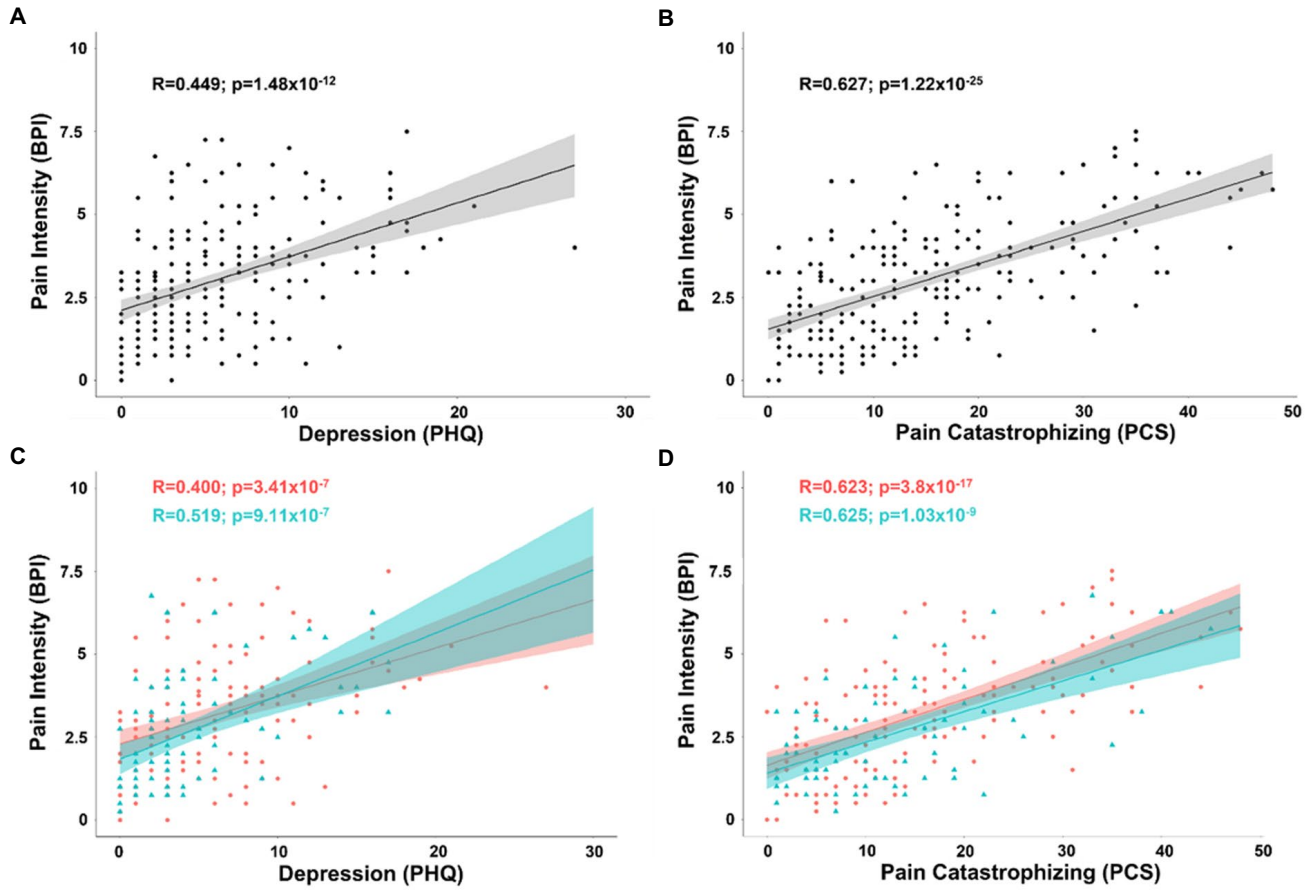
**(A)** Scatterplot displaying the correlation between intensity of chronic pain and depression as measured by the PHQ. **(B)** Scatterplot displaying the correlation between intensity of chronic pain and pain catastrophizing as measured by the PCS. **(C)** Scatterplot displaying the correlation between intensity of chronic pain and depression separated into female (coral/red) and male groups (arctic green/cyan). **(D)** Scatterplot displaying the correlation between intensity of chronic pain and pain catastrophizing separated into female (coral/red) and male groups (arctic green/cyan). Gray, coral, and arctic green shaded areas correspond to 95% confidence curves.

To derive Pearson’s correlation coefficients for covariates associated with pain intensity, we included all continuous and pseudo-continuous covariates of interest without significant collinearity including age, depression (PHQ), pain catastrophizing (PCS), tactile threshold, mechanical pain threshold, area under the curve for temporal summation of mechanical pain, fold change in mechanical pain during temporal summation, pressure pain threshold at the forearm, and pain duration in a partial correlation model. The relationship between pain intensity and depression (*n* = 219; *R* = 0.224; t-stat = 3.32; *p* < 0.001) as well as pain catastrophizing (*R* = 0.520; t-stat = 8.80; *p* < 0.001) both passed BF correction for 45 pairs (*p* ≤ 0.0011). Simple bivariate relationships between pain intensity and depression (*R* = 0.449; t-stat = 7.40; *p* < 0.001) as well as pain catastrophizing (*R* = 0.627; t-stat = 11.9; *p* < 0.001) were also significant (Figures 1A,B).

By separating patients into groups by biological sex, the same model revealed that while pain catastrophizing was significantly associated with pain intensity in both men (*n* = 75; *R* = 0.480; t-stat = 4.41; *p* < 0.001) and women (*n* = 144; *R* = 0.536; t-stat = 7.35; *p* < 0.001), depression showed a similar association in women (*R* = 0.228; t-stat = 2.71; *p* < 0.001), but was no longer a significant covariate with pain intensity in men (*R* = 0.189; t-stat = 1.56; *p* = 0.125). There was no significant difference between men and women in the effect of pain catastrophizing or depression with pain intensity (all tests *p* > 0.05). Simple bivariate relationships between pain intensity and depression (female: *R* = 0.400; t-stat = 5.2; *p* < 0.001; male: *R* = 0.519; t-stat = 5.19; *p* < 0.001) as well as pain catastrophizing (female: *R* = 0.623; t-stat = 9.49; *p* < 0.001; male: *R* = 0.625; t-stat = 6.84; *p* < 0.001) were significant for both women and men (Figures 1C,D).

**Figure 2:**
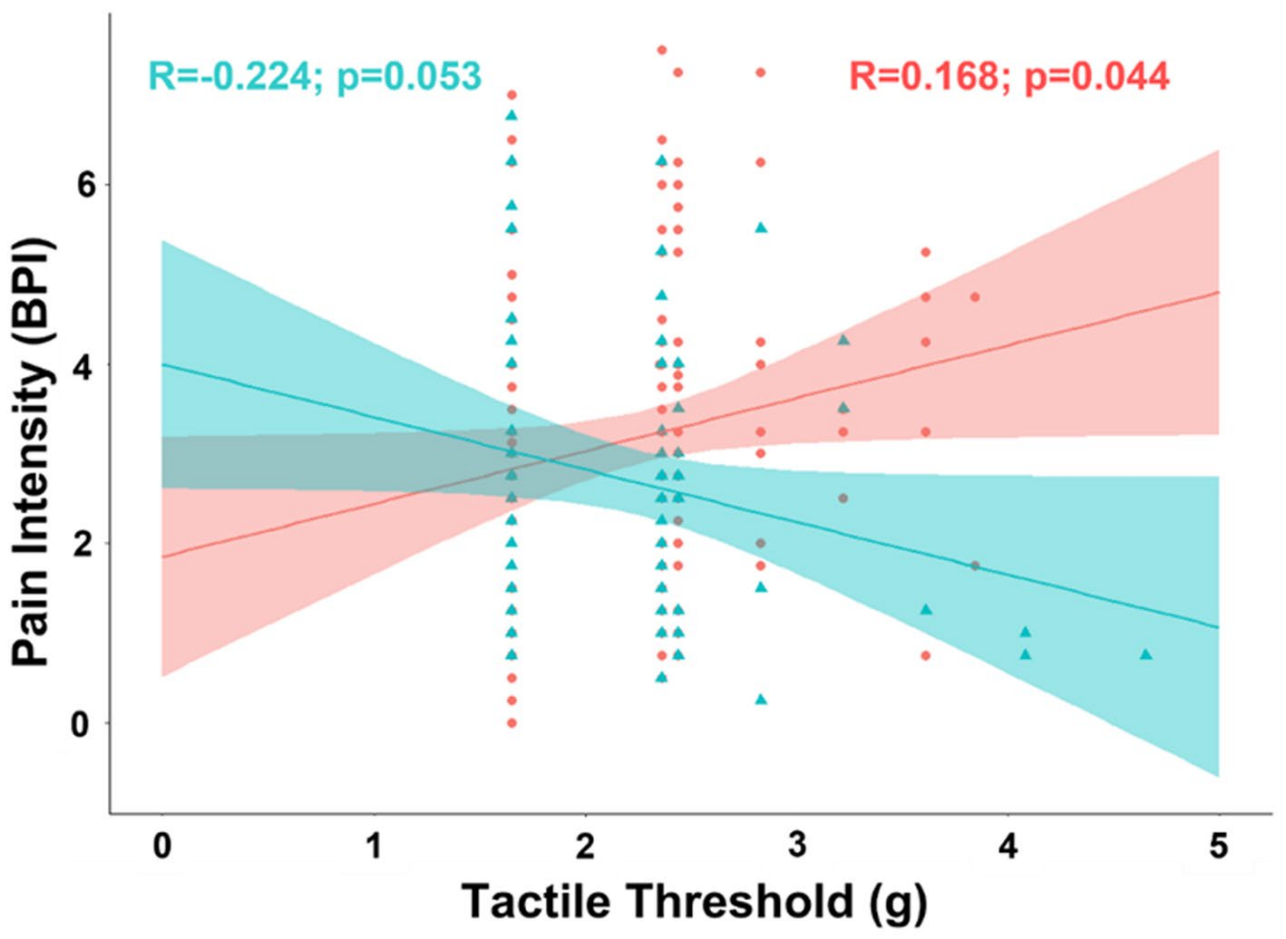
Scatterplot displaying the correlation between intensity of chronic pain and tactile sensitivity separated into female (coral/red) and male groups (arctic green/cyan). Coral and arctic green shaded areas correspond to 95% confidence curves.

Exploratory analyses of biological sex differences in correlation of pain intensity with covariates included in the partial correlation model found tactile threshold was negatively associated with pain intensity in men (*R* = −0.316; t-stat = −2.68; *p* = 0.00928), but not women (*R* = 0.025; t-stat = 0.285; *p* = 0.776). The difference between the correlations between pain intensity and tactile threshold was significant after controlling for factors included in the partial correlation model (pooled *n* = 109; ΔR = 0.341; t-stat = 3.75; *p* < 0.001) and in bivariate correlations (Δ*R* = 0.392; t-stat = 4.41; *p* < 0.001) (Figure 2).

### 3.5. Depression, pain condition, and pain catastrophizing are associated with pain interference and sex differences

For pain interference, being diagnosed with ankylosing spondylitis compared to rheumatoid arthritis (*β* = 5.322, *p* = 0.035), living as a couple compared to living alone (*β* = 8.016, *p* = 0.031), pain catastrophizing (*β* = 0.564, *p* < 0.001), and depressive symptoms (*β* = 1.145, p < 0.001) were significantly positively associated factors. The regression model for examining factors associated with pain interference was significant, *n* = 216, *F*(21, 194) = 9.95, *p* < 0.001, accounting for 46.6% of the variance in the pain interference (Table 3).

The regression model for examining factors associated with pain interference for males was significant, *n* = 74, *F*(20, 53) = 4.39, *p* < 0.001, accounting for 48.2% of the variance in the pain interference. In this model, being younger (*β* = −9.627, *p* = 0.042) and higher pain catastrophizing (*β* = 0.754, *p* = 0.001) was significantly associated with higher pain interference. For female participants, both psychological factors were significantly associated with pain interference, indicating higher depressive symptoms (*β* = 1.361, *p* < 0.001) and pain catastrophizing (*β* = 0.482, *p* < 0.001), which were associated with higher pain interference. The model was significant, *n* = 142, *F*(20, 121) = 6.07, *p* < 0.001, accounting for 41.8% of the variance in the pain interference (Table 4).

**Figure 3:**
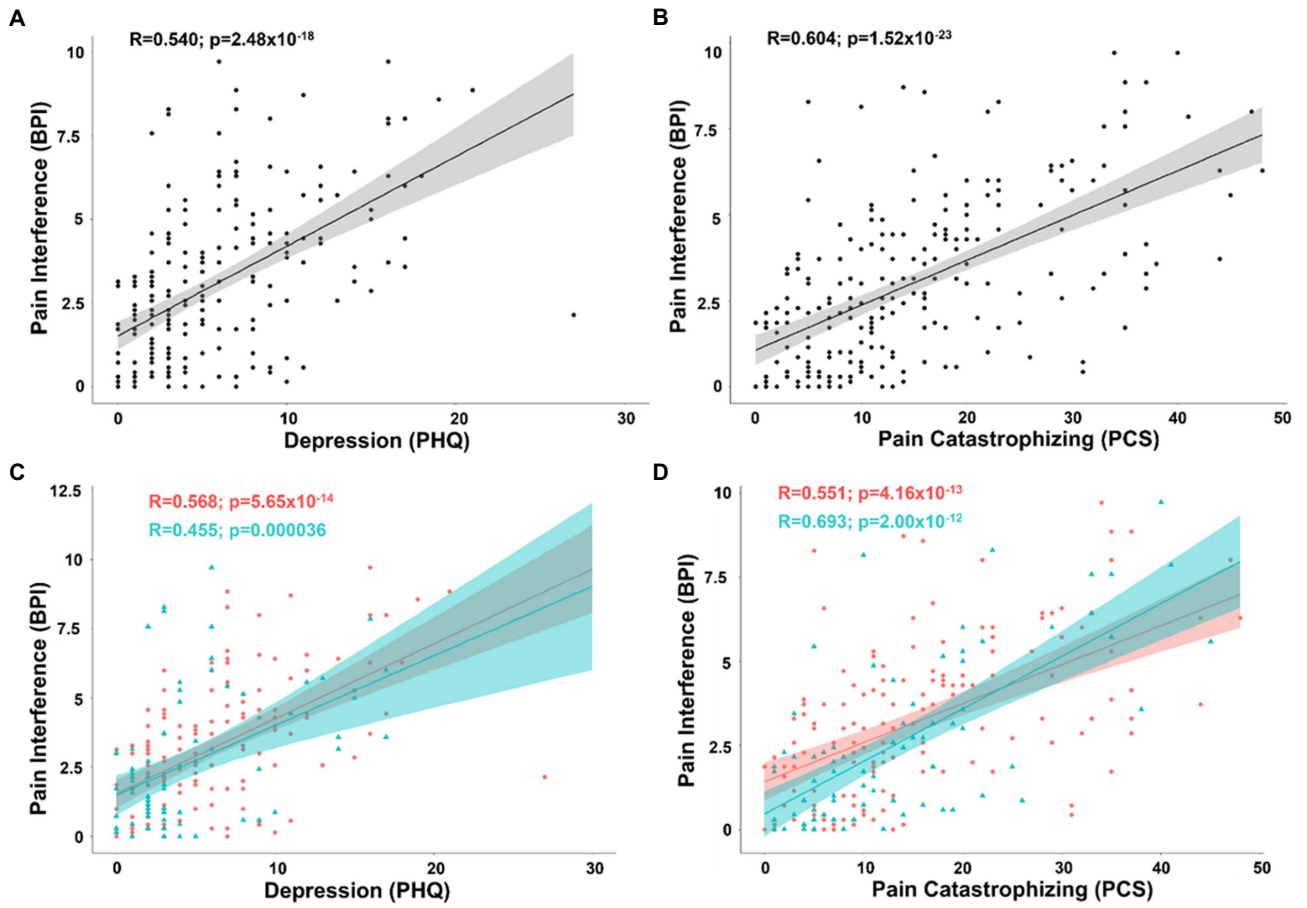
**(A)** Scatterplot displaying the correlation between pain interference and depression as measured by the PHQ. **(B)** Scatterplot displaying the correlation between pain interference and pain catastrophizing as measured by the PCS. **(C)** Scatterplot displaying the correlation between pain interference and depression separated into female (coral/red) and male groups (arctic green/cyan). **(D)** Scatterplot displaying the correlation between pain interference and pain catastrophizing separated into female (coral/red) and male groups (arctic green/cyan). Gray, coral, and arctic green shaded areas correspond to 95% confidence curves.

To derive Pearson’s correlation coefficients for covariates associated with pain inference, we included all continuous and pseudo-continuous covariates of interest without significant collinearity including age, depression (PHQ), pain catastrophizing (PCS), tactile threshold, mechanical pain threshold, area under the curve for temporal summation of mechanical pain, fold change in mechanical pain during temporal summation, pressure pain threshold at the forearm, and pain duration in a partial correlation model. The positive correlations between pain interference and depression (*n* = 219; *R* = 0.351; t-stat = 5.41; *p* < 0.001) as well as pain catastrophizing (*R* = 0.464; t-stat = 7.58; *p* < 0.001) both passed BF correction within the partial correlation model and in bivariate correlations of pain interference and depression (*R* = 0.540; t-stat = 9.47; *p* < 0.001) and pain catastrophizing (*R* = 0.604; t-stat = 11.2; *p* < 0.001) (Figures 3A,B).

Separating patients into groups by biological sex, the same model revealed that while pain catastrophizing was significantly associated with pain interference in both men (*n* = 75; *R* = 0.609; t-stat = 6.20; *p* < 0.001) and women (*n* = 144; *R* = 0.403; t-stat = 5.10; *p* < 0.001), depression showed a similar association in women (*R* = 0.439; t-stat = 5.66; *p* < 0.001), but was no longer a significant covariate with pain interference in men (*R* = 0.022; t-stat = 0.179; *p* = 0.859). There was a significant difference between males and females in the association of depression on pain interference (pooled *n* = 109 Δ*R* = 0.417; t-stat = 4.75; *p* < 0.001). In contrast, in bivariate analysis, while not controlling for confounding variables, pain interference was positively correlated with depression in both men (*R* = 0.455; t-stat = 4.37; *p* < 0.001) and women (*R* = 0.568; t-stat = 8.22; *p* < 0.001) (Figure 3C). To determine the reason for this loss of correlation in the three-variable model between depressive symptoms and pain interference in men, we analyzed the partial correlation after removing pain catastrophizing from the covariance matrix. Removing catastrophizing from the partial correlation model resulted in a positive correlation between pain interference and depression (*R* = 0.439; t-stat = 3.97; *p* < 0.001). This demonstrates that, in this sample of men, the bivariate relationship between pain interference and depression is driven by pain catastrophizing as far as the cross-sectional study design allows us to determine drivers of effects. In women and men, bivariate positive correlations remained between pain interference and catastrophizing (female: *R* = 0.551; t-stat = 7.87; *p* < 0.001; male: *R* = 0.693; t-stat = 8.27; *p* < 0.001) (Figure 3D).

**Figure 4:**
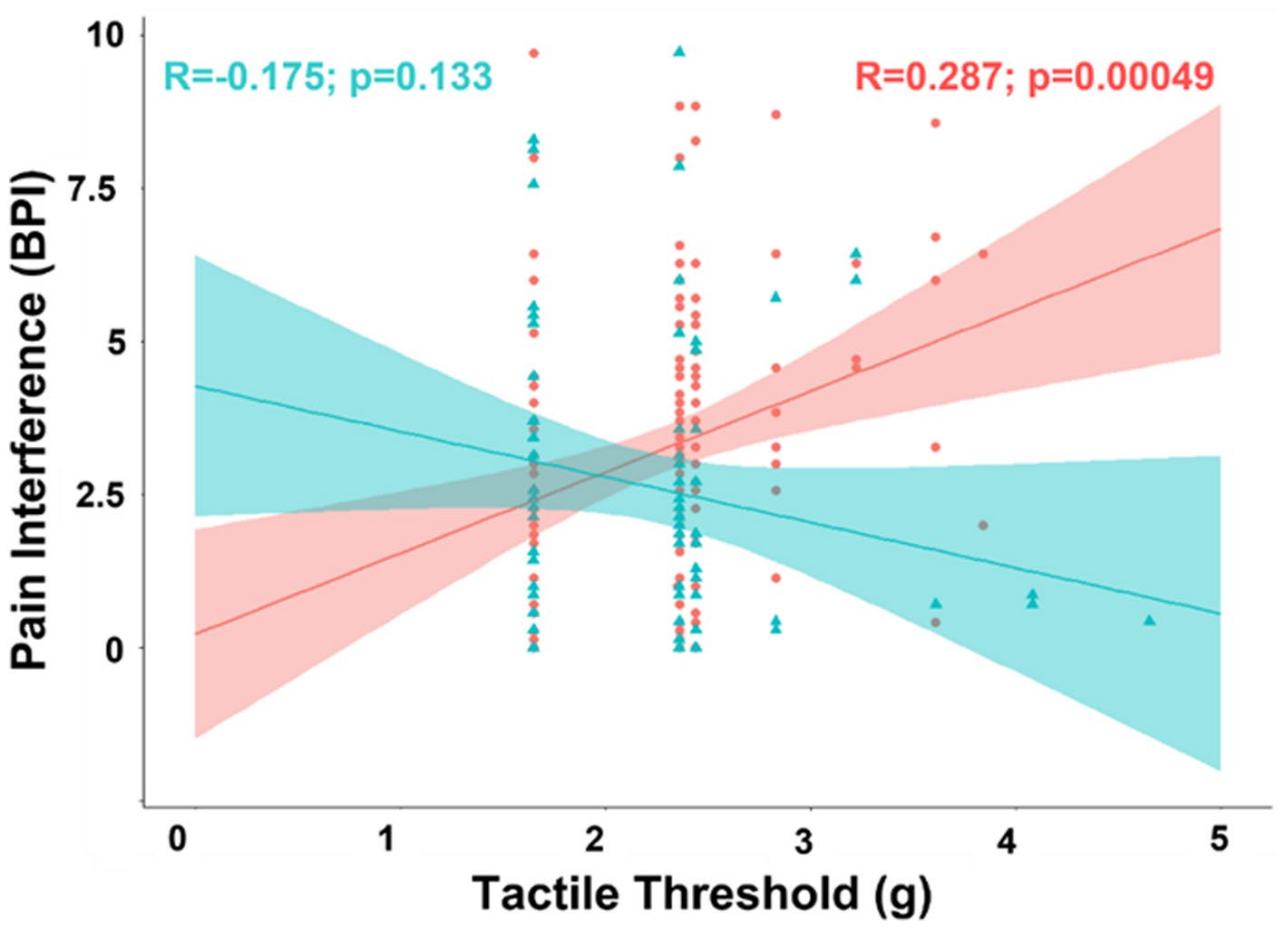
Scatterplot displaying the correlation between pain interference and tactile sensitivity separated into female (coral/red) and male groups (arctic green/cyan). Coral and arctic green shaded areas correspond to 95% confidence curves.

Exploratory analyses of biological sex differences in correlation of pain interference with covariates included in the partial correlation model found tactile threshold was negatively associated with pain interference in men (*R* = −0.253; t-stat = −2.11; *p* = 0.039), but positively correlated in women (*R* = 0.207; t-stat = 2.45; *p* = 0.016). The difference between the correlations between pain interference and tactile threshold was also significant in the partial correlation model (Δ*R* = 0.460; t-stat = 5.36; *p* < 0.001) and in simple bivariate correlations (Δ*R* = 0.462; t-stat = 5.39; *p* < 0.001) (Figure 4).

### 3.6. Depression is associated with number of pain areas reported

**Figure 5:**
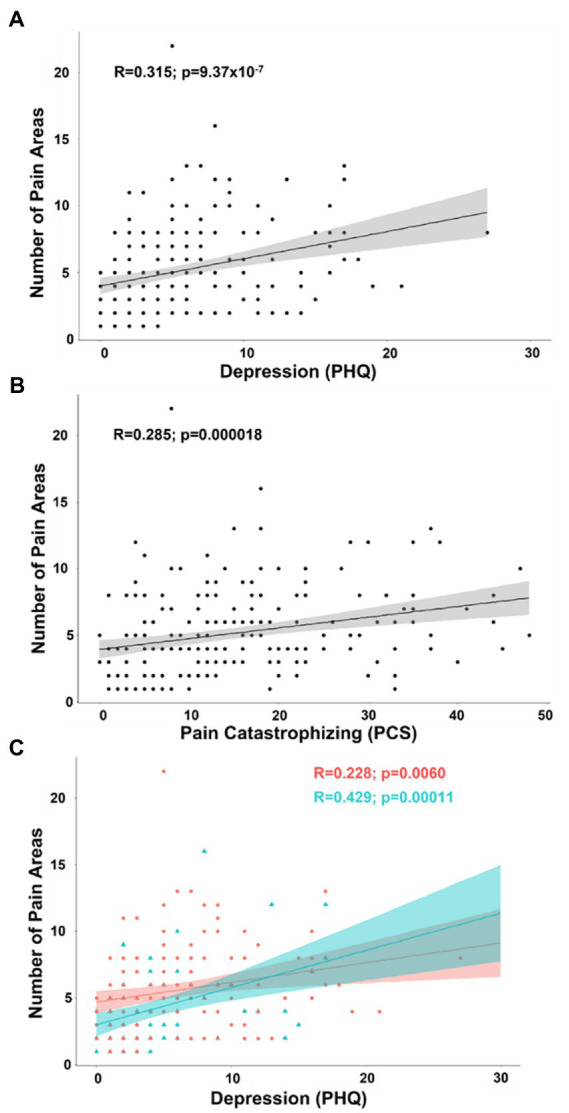
**(A)** Scatterplot displaying the correlation between number of pain areas reported and depression as measured by the PHQ. **(B)** Scatterplot displaying the correlation between number of pain areas reported and pain catastrophizing as measured by the PCS. **(C)** Scatterplot displaying the correlation between number of pain areas reported and depression separated into female (coral/red) and male groups (arctic green/cyan). Gray, coral, and arctic green shaded areas correspond to 95% confidence curves.

In an exploratory analysis of number of pain areas reported, we included all continuous and pseudo-continuous covariates of interest without significant collinearity including age, depression (PHQ), pain catastrophizing (PCS), tactile threshold, mechanical pain threshold, area under the curve for temporal summation of mechanical pain, fold change in mechanical pain during temporal summation, pressure pain threshold at the forearm, and pain duration in a partial correlation model. The relationship between number of pain areas and depression (*n* = 219; *R* = 0.215; t-stat = 3.19; *p* = 0.00165) as well as pain catastrophizing (*R* = 0.136; t-stat = 1.99; *p* = 0.048) were significant in exploratory analysis. In simple bivariate analyses, the relationship between number of pain areas and depression (*R* = 0.315; t-stat = 4.89; *p* < 0.001) as well as pain catastrophizing (*R* = 0.285; t-stat = 4.38; *p* < 0.001) were significant in exploratory analysis (Figures 5A,B). By separating patients into groups by biological sex, the partial correlation model revealed depression was more strongly related to number of pain areas reported in men (*n* = 75; *R* = 0.332; t-stat = 2.74; *p* = 0.0080) than women (*n* = 144; *R* = 0.148; t-stat = 1.73; *p* = 0.085), but this difference was not significant (Figure 5C).

## 4. Discussion and conclusions

This study explored levels of pain intensity and pain interference among Korean patients with chronic secondary musculoskeletal pain in various rheumatic diseases, and its associations with multidimensional factors based on the Biopsychosocial model of pain. The average pain intensity of the current study was 3 on a scale from 0 to 10. This is similar to previous results in an elderly Korean population with chronic pain (Kim et al., 2020). However, this is somewhat lower compared to previous studies among Western populations which ranged from 6 to 8 in the US (Farrar et al., 2001; Smit et al., 2020; Zvolensky et al., 2020), in Spain (Bodes Pardo et al., 2018), and Sweden (Grimby-Ekman et al., 2020) in patients with chronic pain. This is supported by previous reports of underreporting and underdiagnosis of pain in Asians (Kawi et al., 2019). Cultural norms of Asian patients may discourage reporting their pain to avoid burdening others or being seen as weak (Tung and Li, 2015). Rather than seeking medical assistance, accepting the pain as a natural thing (Im et al., 2008) or suffering to maintain one’s independence (Dubus, 2014; Nguyen and Seal, 2014) has been reported in Asian immigrants in the US. The participants of the current study were recruited from a clinical setting in an outpatient clinic in a hospital, and most had experienced pain for more than 5 years. Previously, higher pain sensitivity has been reported in Asians compared to non-Hispanic Whites (Kim et al., 2017). The discrepancy of these results in pain intensity reports may be associated with the cultural background of the Korean population in the way they express their pain. Cultural aspects (e.g., stereotyped stoicism; Buddhism) and social norms embedded in Asian cultures may influence patients’ pain perception and their expression of pain intensity (Good and Ahn, 2008; Ahn et al., 2021). Therefore, careful attention may be required when assessing pain experience, and multidimensional assessment may mitigate this potential predisposition for under-reporting pain in this specific ethnic group when dealing with chronic secondary musculoskeletal pain.

Among the multidimensional factors associated with pain intensity and pain interference, we found several significant factors for each biological, psychological, and social measures. Among these three groups of factors, psychological factors (depressive symptoms and pain catastrophizing) were the most influential in both pain intensity and pain interference when compared to biological and social factors. Depressive symptoms (Zvolensky et al., 2020; Kim et al., 2021) and pain catastrophizing (Bodes Pardo et al., 2018; Kamonseki et al., 2021; Kim et al., 2021) are well known factors associated with chronic pain, and this study adds to the literature by providing additional evidence of the significance of these factors amongst other factors, specifically in this cultural group. About half of the participants in this study had some level of depressive symptoms, and this is comparable with a previous study in Koreans (Kim et al., 2020). We also found the significant mediating role of pain catastrophizing on the association between depression and pain interference among male participants. This result is in line with previous study on elderly Korean Americans with chronic pain (Kim et al., 2021). We suggest including these psychological factors in pain assessment in a Korean population to capture a more complete picture of their pain experience and to improve outcomes of pain management.

Biological sex is a well-known factor influencing chronic pain (Mogil and Bailey, 2010); we add to the literature by reporting sex differences on the impact of pain catastrophizing and depression on pain. For example, depressive symptoms were significantly associated with pain intensity and pain interference only for women, while there were no significant associations in men, including all other biopsychosocial factors in the model. Also, for men, being young (compared to those higher than 60 years old) was significantly associated with higher pain interference. These differences should inform healthcare providers to consider these factors for developing not only culturally adapted, but also sex-specific, programs targeted to reduce levels of pain intensity and pain interference.

Interestingly, measures of QST did not specifically contribute to explaining pain intensity or pain interference in the multivariable analyses. However, we found some sex differences in the correlational analyses, where tactile threshold was negatively associated with pain interference in men but positively correlated in women. This is a novel sex difference in the relationship between tactile sensitivity and pain intensity and interference and needs to be followed up and replicated. The source of the parallel finding that pain intensity and pain interference are both related to tactile sensitivity in a consistent manner in men and women is that pain intensity and pain interference are highly correlated (Cano et al., 2006; Fullwood et al., 2021; Kim et al., 2021). Several studies have demonstrated that both acute and chronic pain reduces tactile sensitivity and tactile acuity (Seltzer and Seltzer, 1986; Bolanowski et al., 2001; Magerl and Treede, 2004; Drummond and Blockey, 2009; Adamczyk W. et al., 2018; Adamczyk W.M. et al., 2018). Tactile sensitivity is directly related to tactile acuity (Craig and Kisner, 1998; Gibson and Craig, 2002; Zingaretti et al., 2019). Studies have shown that reductions in tactile acuity, measured as two-point discrimination, are related to the intensity of chronic pain. These behavioral changes have been linked to evidence of neuroplastic changes in the somatosensory cortex and somatosensory thalamus associated with chronic pain (Kong et al., 2013; Kim et al., 2020). On a group level, changes in tactile acuity are largely consistent across studies, however, efforts to train individuals with chronic pain in tactile discrimination as an analgesic intervention have not achieved consistent analgesia (Moseley et al., 2008; Ryan et al., 2014; Adamczyk W. et al., 2018; van Baal et al., 2020). Our findings suggest that there may be an important sex difference in how tactile acuity is related to chronic pain intensity and interference that has previously been ignored in these interventional studies.

While previously it has been reported that experimental pain sensitivity was associated with pain intensity (Bagnato et al., 2015; Amiri et al., 2021), besides the tactile sensitivity, we did not find any significant relationships between experimental pain sensitivity and clinical pain intensity or interference. The average PPT was 8.12 Nm, which is somewhat higher compared to previous studies in chronic pain patients (Bodes Pardo et al., 2018; Amiri et al., 2021). The results imply low levels of sensitivity in the current study participants, and this is in contrast with previous reports (Kim et al., 2017). It is difficult to explain these results, but we did not include the full modality (e.g., heat pain or cold pain) of experimental pain sensitivity in our study. An additional possible explanation is that participants were new to the QST procedure and showed difficulty in reporting their pain perception. We would suggest more studies with more extensive and rigorous QST measurement in this population. However, it must be cautioned that while QST has been shown to correlate with pain symptomatology and intensity, large studies have not demonstrated that QST predicts development of chronic pain (Slade et al., 2014). The usefulness of QST may be to diagnose subtypes of chronic pain, but not to usefully anticipate overall pain intensity or interference (Shraim et al., 2022).

This study has several limitations. First, we included multiple measures to build a comprehensive model explaining chronic pain, however, there are still other important factors that we did not include (e.g., anxiety, social support, quality of life, kinesiophobia, pain acceptance, and coping strategies) that have been reported previously (Esteve et al., 2007; Varallo et al., 2022). Also, participants were recruited from an outpatient center at a hospital which implies the participants were receiving medical care for their diseases. Therefore, the results of the current study may not be generalized to those in the community without appropriate chronic pain care. Finally, because of the nature of study design, associations reported in this study cannot be interpreted as causal relationships.

The current study explored biopsychosocial correlates associated with pain intensity and pain interference among patients with chronic secondary musculoskeletal pain. Pain catastrophizing had the greatest influence on pain experience for both male and female patients, and depressive symptoms were associated with pain intensity and pain interference among female patients. These identified factors should be included in pain assessment and pain management for patients with chronic pain. A sex-specific approach would be warranted to better manage patients with chronic secondary musculoskeletal pain in various rheumatic diseases. Clinicians and researchers who serve diverse racial/ethnic groups should consider the significant role of cultural and psychological factors in understanding chronic pain experiences, specifically for Asian populations.

## Data availability statement

The raw data supporting the conclusions of this article will be made available by the authors, without undue reservation.

## Ethics statement

The studies involving human participants were reviewed and approved by the Ajou University Hospital IRB (AJIRB-MED-SUR-21-319). The patients/participants provided their written informed consent to participate in this study.

## Author contributions

HK and TM designed and conceptualized the study, analyzed the data, and drew all the charts. HK, J-YJ, J-WK, and HK collected the data. HK, TM, and H-AK organized the data. HK organized the whole data collection process. H-AK supervised the quality of the data collection. HK wrote the first draft of the article. All authors participated in the revision of the manuscript and approved the final manuscript for publication.

## Funding

This study was supported by the National Research Foundation of Korea (NRF) grant funded by the Korea government (MSIT) (no. 2021R1F1A1059877) and the National Institute of General Medical Sciences (RL5GM118972).

## Conflict of interest

The authors declare that the research was conducted in the absence of any commercial or financial relationships that could be construed as a potential conflict of interest.

## Publisher’s note

All claims expressed in this article are solely those of the authors and do not necessarily represent those of their affiliated organizations, or those of the publisher, the editors and the reviewers. Any product that may be evaluated in this article, or claim that may be made by its manufacturer, is not guaranteed or endorsed by the publisher.
